# Waste-Based One-Part Alkali Activated Materials

**DOI:** 10.3390/ma14112911

**Published:** 2021-05-28

**Authors:** Margarida Gonçalves, Inês Silveirinha Vilarinho, Marinélia Capela, Ana Caetano, Rui Miguel Novais, João António Labrincha, Maria Paula Seabra

**Affiliations:** 1 Department of Environment and Planning, Campus Universitário de Santiago, University of Aveiro, 3810-193 Aveiro, Portugal; 2 Department of Materials and Ceramic Engineering/CICECO—Aveiro Institute of Materials, Campus Universitário de Santiago, University of Aveiro, 3810-193 Aveiro, Portugal; inessvilarinho@ua.pt (I.S.V.); marinelia.capela@ua.pt (M.C.); anacaetano@ua.pt (A.C.); ruimnovais@ua.pt (R.M.N.); jal@ua.pt (J.A.L.)

**Keywords:** one-part, alkali activated materials, blast furnace slag, bottom bed ashes, compressive strength

## Abstract

Ordinary Portland Cement is the most widely used binder in the construction sector; however, a very high carbon footprint is associated with its production process. Consequently, more sustainable alternative construction materials are being investigated, namely, one-part alkali activated materials (AAMs). In this work, waste-based one-part AAMs binders were developed using only a blast furnace slag, as the solid precursor, and sodium metasilicate, as the solid activator. For the first time, mortars in which the commercial sand was replaced by two exhausted sands from biomass boilers (CA and CT) were developed. Firstly, the characterization of the slag and sands (aggregates) was performed. After, the AAMs fresh and hardened state properties were evaluated, being the characterization complemented by FTIR and microstructural analysis. The binder and the mortars prepared with commercial sand presented high compressive strength values after 28 days of curing-56 MPa and 79 MPa, respectively. The mortars developed with exhausted sands exhibit outstanding compressive strength values, 86 and 70 MPa for CT and CA, respectively, and the other material’s properties were not affected. Consequently, this work proved that high compressive strength waste-based one-part AAMs mortars can be produced and that it is feasible to use another waste as aggregate in the mortar’s formulations: the exhausted sands from biomass boilers.

## 1. Introduction

Ordinary Portland Cement (OPC) is the most widely used binder in the construction sector being the basic component of mortars and concrete. However, OPC production contributes to, approximately, 6% of the total anthropogenic global CO_2_ emissions [1] and it is obtained by a high energy demanding process, consuming about 20% of the European Union industrially used energy [2]. Consequently, it is necessary to develop more sustainable alternative construction materials. In this context, the alkali-activated materials (AAMs) arise as a lower carbon footprint alternative provided that careful mixture design is employed in their synthesis, which can result in a carbon emission reduction of up to 50% [3]. AAMs are commonly produced by mixing alumino-silicate materials (e.g., metakaolin) and an alkaline solution (e.g., sodium hydroxide, sodium silicate). Additionally, eco-AAMs have been developed using industrial wastes such as fly ash (FA) and granulated blast furnace slag (BFS) [4]. These materials present excellent properties, namely, high mechanical strength, heat resistance, low creep, and shrinkage [5]. However, the use of corrosive and viscous alkaline solutions imposes practical constraints in the scale-up of these solutions [6]. Therefore, it is essential to develop new AAM processing methodologies, and one-part AAMs, also known as the “just add water” AAM, appear to be a very promising solution. In this strategy, the precursors and the alkaline activators are used in the solid form (usually as powders) and water is added to start the geopolymerization process [7,8]. The process has obvious similitudes with the preparation of OPC-based materials.

A recent review [9] details the raw materials, alkaline activators, additives, and curing conditions that can be used to prepare one-part AAMs. Relevant physical and mechanical properties, the nature of hydration products on the produced specimens, and their environmental impacts are also discussed. Some published works report the partial replacement of OPC by BFS, bentonite, metakaolin, or fly ash-called hybrid one-part AAMs [10,11,12]. Abdollahnejad et al. [10] prepared mortars with 40 wt.% of OPC, with kaolin, fly ash, and calcium hydroxide being the other components/binders. The obtained samples did not show signs of efflorescence and, after 28 days, the compressive strength values were between 20 and 40 MPa.

The literature also reports the development of one-part AAMs without OPC. The used precursors were wastes or by-products [5,6,13], with coal fly ash [14,15] and BFS [16] being the most common ones. Sturm et al. [17] utilized rice husk ash and obtained very promising results: After the third curing day, the samples exhibited a compressive strength of 33 MPa and, according to the X-ray powder diffraction (XRD) results, a high degree of reaction. Longer curing periods triggered a slight decrease in the mechanical strength (30.1 MPa after 7 days curing); however, no significant changes were observed in XRD and Fourier transform infrared spectroscopy (FTIR) results. Suwan and Fan [18] compared the behaviour of AAMs synthetized by the two processes (traditional and one-part) using fly ash as a precursor. They observed that the one-part process releases a substantial amount of heat while mixing and thus, more water is required to compensate the evaporated water. The compressive strength of the specimens prepared by the traditional method was 17% higher, suggesting that the reaction was more extensive. Several works [9,19] reported, for one-part AAMs, problems of workability and short setting time, which might be solved by adding superplasticizers and setting retarders. Oderji et al. [20] studied the influence of the water/binder ratio (W/B), additives type, and amount (2 to 8 wt.%) on the fresh and hardened state properties of one-part AMMs. As expected, higher W/B ratios extended the setting time of the samples and increased the pastes’ workability (optimal W/B ratio of 0.3); at the same time, the mechanical properties tend to deteriorate. The addition of borax (setting retarder) improved the fresh state properties of the paste, also leading to an increase in the compressive and flexural strengths. According to the authors [20], 6 wt.% of borax is the suitable amount since it improved the paste fluidity without significantly affect the mechanical properties of the samples. Bong et al. [21], besides testing the effect of additives, studied two activators (as powder): Anhydrous sodium metasilicate and sodium silicate. The authors observed that the workability and setting time of the AMMs mixtures prepared with sodium silicate did not fill the requirements, even when additives were used, and a decrease of the compressive strength was observed. On the contrary, in the mixtures prepared with anhydrous sodium metasilicate, it was possible to adjust the pastes’ workability and the setting time leading to interesting mechanical properties (37–45 MPa).

In recent years, the number of natural resources used in construction has increased exponentially, with sand and gravel being the most extracted group of materials worldwide [22]. Although sand is a common resource, its scarcity is already a major issue in cities that are expanding rapidly. Gholampour et al. [23] investigated the substitution of natural sand by wastes (lead smelter slag and glass sand) in conventional geopolymeric mortars, with the compressive strength values being promising, up to 74 MPa.

FA or a mixture of FA and BFS are the solid precursors reported in the literature for the preparation of one-part AAMs, and this work investigates the use of only BFS, aiming to produce a binder with high compressive strength and adequate workability. For the first time, one-part AAMs mortars were developed using a waste as aggregate, more specifically exhausted sands from biomass burning fluidized bed boilers. Relevant fresh and hardened state properties of the binders and mortars were evaluated.

## 2. Materials and Methods

### 2.1. Materials

Blast Furnace Slag (BFS, Ecocem, Aix-en-Provence, France) with strong amorphous character in the as-received condition was used as solid precursor and granular powder of sodium metasilicate (Na_2_O = 47–49.5 wt.%, SiO_2_ = 50.5–53 wt.%, 122.06 g/mol, Sigma-Aldrich, St. Louis, MS, USA) was used as activator. In mortar formulations, three types of sand were studied as aggregates: A calibrated commercial natural siliceous sand (Weber Saint-Gobain, Aveiro, Portugal) hereinafter referred to as W; and two wastes collected from fluidized bed biomass boilers supplied by The Navigator Company (Aveiro, Portugal), named CA and CT. These residues underwent a sieving pre-treatment, and only the granulometric fraction between 2 mm and 63 µm was used (≈85 wt.% of the total residue produced).

### 2.2. Preparation of Alkali-Activated Materials

One-part AAM binder and mortars using three distinct sands (W, CA, and CT) were prepared, and the tested formulations are shown in Table 1. Binder formulations are named B, while M refers to mortars. In these, the second letter identifies the type of aggregate while the number represents the water/binder (W/B) ratio. In this work, the influence of the water amount on the AAMs was studied and adjusted to obtain pastes with the appropriate workability. Preliminary tests were performed to evaluate the impact of the precursor/activator ratio (ranging from 2 to 20) on the samples’ compressive strength. These tests showed that the optimum ratio for the binder formulation was 10; as a result, this ratio was employed in the present investigation.

One-part AAMs were prepared under ambient conditions, using the following procedure:

1. Homogenization of the solid components (precursor and activator), by mixing in a plastic bag for approximately 1 min;
2. Mechanical mixing for 1 min, while adding water;
3. Manually mixing for approximately 1 min;
4. Mechanical mixing for 1.5 min.

For hardened-state characterization, the slurry was cast into 4 × 4 × 4 cm^3^ metallic moulds, vibrated for 2 min using an electric vibrator, and then covered with a plastic film. After 24 h, all samples were demoulded and left at ambient conditions until the 7th and 28th curing day.

### 2.3. Characterization Techniques

#### 2.3.1. Raw Materials Characterization

X-ray fluorescence (XRF, Philips X’Pert PRO MPD spectrometer, Amsterdam, The Netherlands) was used to determine the chemical composition of the materials and the loss on ignition (LOI) at 1000 °C. The aggregate density and water absorption were evaluated according to the standard NP EN 1097-6:2003 [24]. The material’s particle size distribution and microstructure were obtained by laser diffraction in a Coulter LS analyser (LS 230, Fraunhofer optical model, Beckman Coulter, CA, USA) and by scanning electron microscopy (SEM, Hitachi S4100, 25 kV acceleration voltage, Tokyo, Japan), respectively. The particle size distributions of the sands were obtained by sieving, following the standard NP EN 933-1 [25].

#### 2.3.2. One-Part Alkali-Activated Materials Characterization

In the fresh state, flow table tests were performed to assess the workability of the mixtures according to EN 1015-3 [26], while the initial and final setting times were determined using the *Vicat* apparatus, according to EN 196-3 [27]. Calorimetric measurements were carried out to control the temperature evolution of the binder, during the first 24 h of curing, under controlled relative humidity (95%) and temperature (20 °C). These conditions were chosen to represent, as close as possible, the conditions present in the plastic film in which the samples were involved during the first day of curing. Three samples were tested, and the average data are reported.

The hardened state properties were evaluated after 7 and 28 days of curing. The apparent density of samples was determined from their weight and geometric volume. The compressive strength was measured using a Universal Testing Machine (AG-25TA-Shimadzu, Kyoto, Japan) with a displacement rate of 0.5 mm/min, following the standard EN1015-11 [28]. The water absorption of the specimens was measured after 24 h immersion in water, while the coefficient due to capillary action was determined based on the EN1015-18 standard [29]. XRD was used to evaluate the mineralogical composition of the samples, using a Rigaku Geigerflex D/max-Series instrument (Tokyo, Japan), and phase identification by PANalytical X’Pert HighScore Plus PRO3 software (Almelo, The Netherlands). The microstructural analysis was performed with SEM using the equipment identified in the previous section. Attenuated total reflection Fourier transform infrared spectroscopy (FTIR-ATR) was carried out in powdered samples (manually disaggregated) with a IFS FTIR spectrophotometer (Brucker, Billerica, MA, USA) equipped with a single horizontal Golden Gate diamond ATR cell. The data were obtained with a 4 cm^−1^ resolution, with 256 scans per spectrum in a range between 4000–400 cm^−1^.

## 3. Results and Discussion

### 3.1. Characterization of the Materials

The chemical composition of BFS, as well as sands, was determined by XRF, and the components are shown in Table 2. CaO is the primary component of BFS (46.55 wt.%), with SiO_2_ and Al_2_O_3_ being the other major constituents (32.43 and 9.21 wt.%, respectively). Their total corresponds to 90 wt.% of the composition, confirming the requirement of the pozzolanic material and the adequacy for geopolymerization [30]. The high content of CaO decreases the setting time since when react with water generates heat from the exothermic process, accelerating the reaction rate [31,32]. SiO_2_ is the most abundant oxide in the sands (97.15, 64.57, and 80.80 wt.%, for W, CA, and CT respectively), but the waste sands also show a significant amount of calcium (20 wt.% CA; 7.3 wt.% CT). Another noteworthy factor is the difference in LOI values in CA and CT compared to the reference sand, probably due to its chemical composition (presence of CaCO_3_ and/or organic matter).

The three tested aggregates exhibit similar densities (2.68, 2.62, and 2.64 g/cm^3^, respectively, for W, CA, and CT). The water absorption of commercial sand (W) is 0.049%. The waste sands have slightly higher values (0.293 and 0.070%, for CA and CT, respectively).

**Figure 1:**
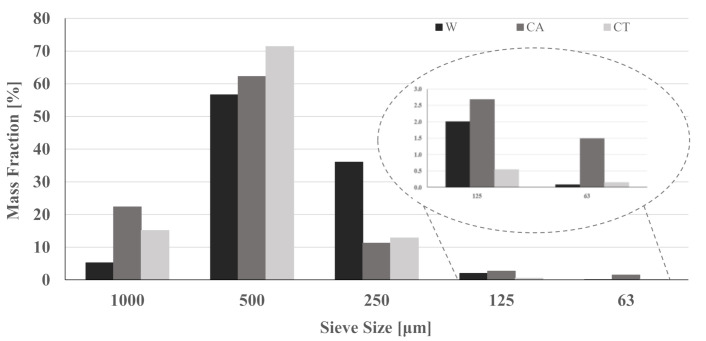
Granulometric distribution of the aggregates: Commercial sand (W) and waste sands (CA and CT).

**Figure 2:**
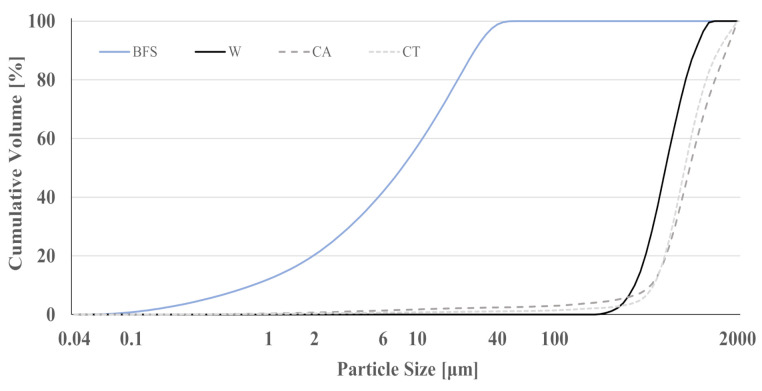
Particle size distribution of BFS and aggregates: W, CA and CT.

Figure 1 shows the particle size distribution of the as-received sands. The highest mass content was obtained for the fraction with an opening of 500 μm (comprising 57, 62, and 71% of the total mass of W, CA, and CT sands, respectively). Waste sand CA presents finer particles, with ≈4 wt.% of the mass retained in the narrower sieves (<125 μm). As previously mentioned, the waste sands were sieved and only the fraction between 2 mm and 63 µm was used. The resulting particle size distribution is given in Figure 2, together with that of BFS and W sand. The reference sand shows narrower and more uniform grain size distribution (mean particle size of 625 μm). Despite the sieving step, the waste sands still show coarser particles compared to the commercial sand with the mean particle size being 830 and 900 μm for CT and CA, respectively. BFS has a broader particle size distribution but is composed of much finer grains (average value of 8 μm), as required for a reactive precursor.

**Figure 3:**
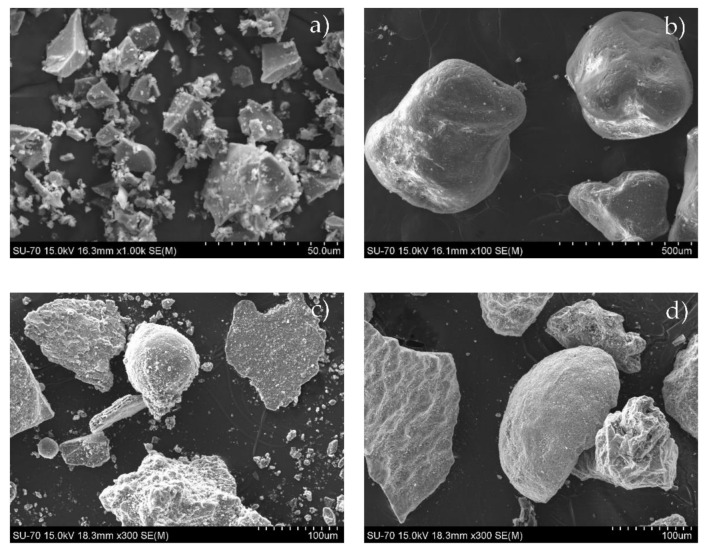
SEM micrographs of the BFS (**a**) and sands: W (**b**); CA (**c**); CT (**d**).

The particle morphology of BFS and sands is illustrated by the SEM micrographs in Figure 3. BFS particles have irregular shapes (Figure 3a). The W sand particles have more regular rounded forms (Figure 3b), while the waste sands are composed of more irregular grains (Figure 3c,d).

### 3.2. Binder and Mortars Characterization

#### 3.2.1. Fresh State Characterization

**Figure 4:**
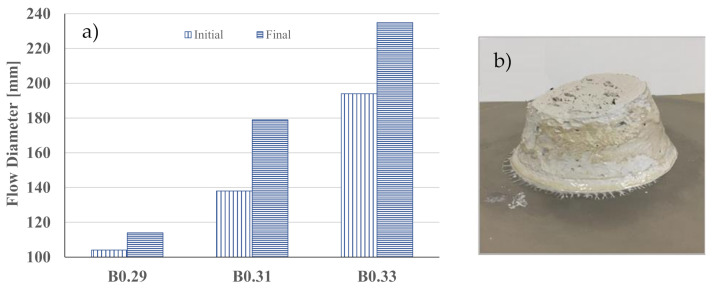
Flow diameters for binders (**a**) and final appearance of B0.29 mixture (**b**).

**Figure 5:**
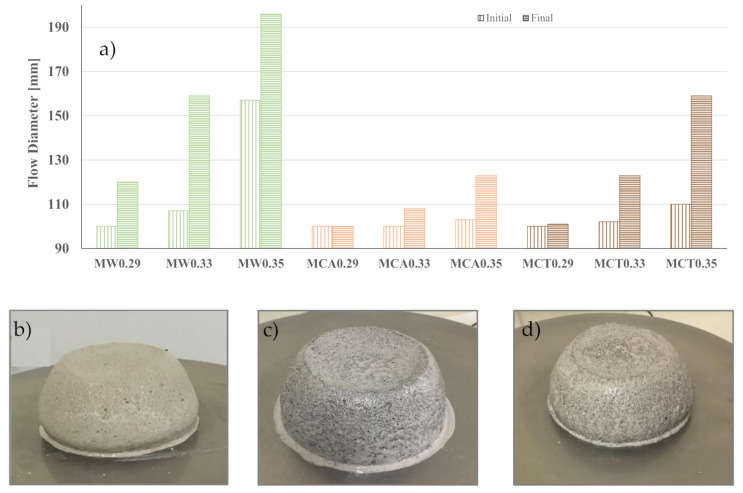
Flow diameters for mortars (**a**) and final appearance of MW0.29 (**b**) MCA0.35 (**c**) and MCT0.33 (**d**) mortars.

The workability of the mixes (Table 1) was firstly evaluated to optimize the water/binder (W/B) ratio. Figure 4 and Figure 5 show the results of the flow table test, respectively, for binders and mortars, as well as the appearance of adjusted mixtures at the end of the test. As expected, the greater the amount of water in the mixture, the higher the flowability. In the binder, the increase of W/B from 0.29 to 0.31 and 0.33 induces an increase of 57 and 106%, respectively, in the final flow diameter.

**Figure 6:**
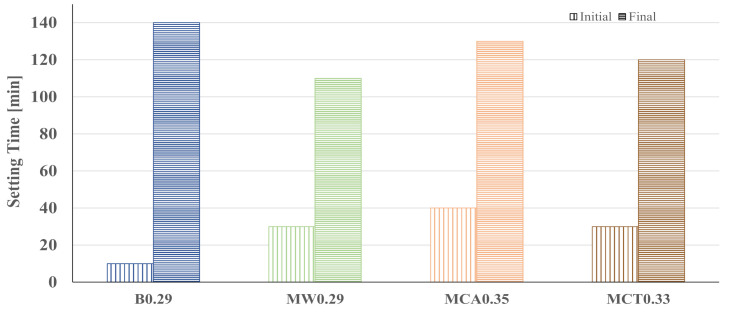
Initial and final setting times for optimized binder and mortars.

The same trend is noticed on mortars (Figure 5a), but the magnitude of the effect depends on the type of sand used in the formulations. With commercial sand (MW mixes), the change on W/B causes a strong impact on the spread values, meaning that binder action is dominating. The commercial sand is chemically inert, and the rounded shape of the particles also contributes to this behaviour [33]. The spread of MCA and MCT mortars is less affected by the W/B variations, particularly the first ones. These materials remain sticky, and the change of W/B from 0.29 to 0.35 induces a gain of only 20 mm in the final spread. CA sand contains a significant amount of CaO (see Table 2) and is composed of finer particles, and both factors might enhance its interaction with the water. This effect is also noticed in MCT samples but in a lower degree. Assuming 120 mm as the optimal final spread, the commercial sand needs a W/B ratio of 0.29, but higher ratios are required by the waste-based sands, namely, 0.33 (for CT sand) and 0.35 (for CA sand). Thus, henceforward, the optimized samples in terms of water content/workability are B0.29, MW0.29, MCA0.35, and MCT0.33. Based on the aforementioned considerations, the initial and final setting times were measured only for the optimal formulations and the results are shown in Figure 6.

The binder shows initial and final setting times of 10 and 140 min, respectively. These values seem reasonable for real applications and do not require retarders or accelerators. Mortars tend to show a delay in the beginning of setting, due to the presence of aggregates and/or the use of higher water content. This is more evident with MCA0.35 (40 min) due to the presence of finer particles that required an extra amount of water, but the prolonged setting can be a practical advantage in in-situ applications. The impact of the nature of the aggregate is less pronounced on the slurries final setting, the values ranging from 110 to 130 min. When compared with mortars and as expected, B0.29 has a longer final setting time, since it has a higher water content and more material to react.

**Figure 7:**
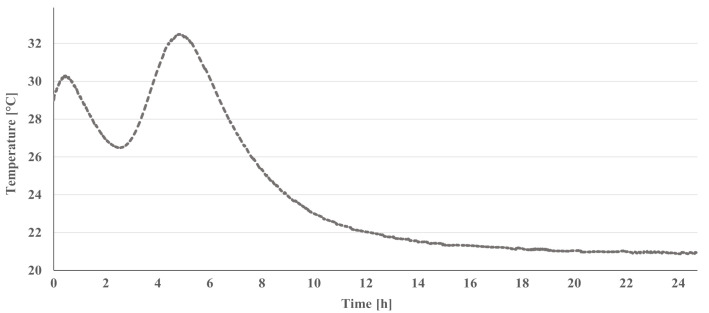
Temperature evolution during the first 24 h of curing for B0.29 formulation.

To evaluate the behaviour of the binder in the alkali-activation kinetics, calorimetric measurements were performed with the optimized formulation B0.29. The temperature evolution upon the first 24 h of curing is shown in Figure 7.

Looking at Figure 7, it is visible that B0.29 presents a first peak (pre-induction period) about 25 min after the beginning of the reaction and represents the wetting and dissolution of the solid activator. The second peak (acceleration/deceleration period), whose maximum is reached about 5 h after the start, corresponds to the nucleation, growth, and precipitation of the reaction products [34]. In addition, compared with conventional AAMs [35,36], the results indicate that the reaction in the one-part binder is faster at 16 vs. 20 h [35] and the temperature increase is also higher at 4 vs. 2 °C [36]. However, the results are very similar to those obtained for the one-part mortar developed by Luukkonen et al. [37].

#### 3.2.2. Hardened State Characterization

**Figure 8:**
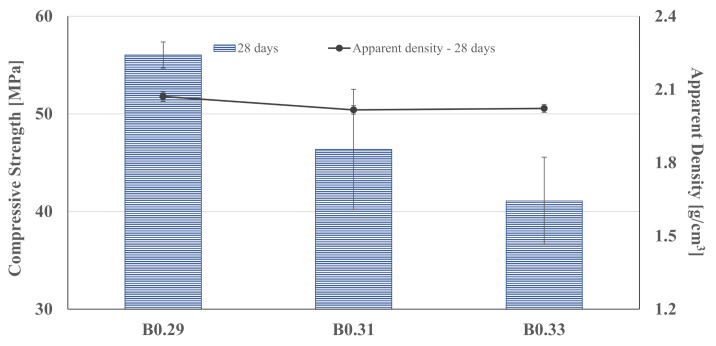
Compressive strength and apparent density of the binders, at 28 days of curing.

The compressive strengths of the studied binders, as well as their apparent density at 28 days of curing, are shown in Figure 8.

The characterization revealed that the apparent density of the developed binders is very similar. As expected, the compressive strength of the samples decreases with the W/B ratio increase. Analyzing the values, it is observed that by increasing the W/B ratio from 0.29 to 0.31 and 0.33, the compressive strength decreases by 17% and 27%, respectively. This fact is explained by the increase in water content, where the excess of water (not used for the geopolymerization reaction) evaporates and leads to a higher percentage of air voids.

**Figure 9:**
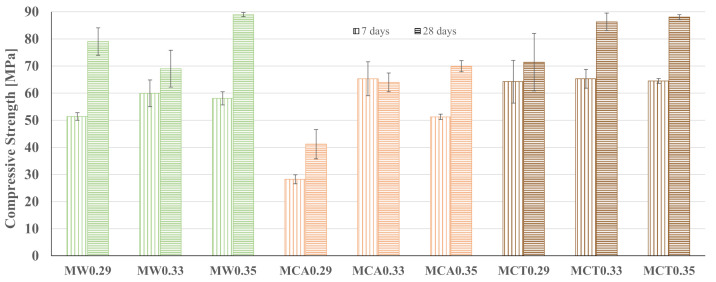
Compressive strength of the mortars at 7 and 28 days of curing.

The apparent density and the compressive strength of the mortars, at 7 and 28 days of curing, are shown in Table 3 and Figure 9, respectively.

For the different mortars, the apparent density obtained also remains practically constant with age. It is clear that samples with CA sand are less dense and MW mortars have higher density values, as a result of differences in their granulometric distribution. Compressive strength, on the other hand, follows the opposite trend to that observed in binders. However, it is important to consider that for mortars, additional water was necessary not only for the binder reaction but also to involve and lubricate the sand particles and get a homogeneous mixture [38].

The compressive strength of the mortars containing the W sand, after 28 days of curing, varies between 70 and 90 MPa depending on the W/B ratio. For mortars with the waste sand CA, it can be immediately depicted that the W/B ratio of 0.29 was not enough for the geopolymerization reaction, reaching the samples only a strength of 40 MPa. With the rise of the W/B ratio to 0.33 and 0.35, an increase of 55 and 70% on the compressive strength was achieved, respectively. The need for a higher water amount can be explained by the smaller grain size of CA and, therefore, a larger specific surface area, so a higher water content is required to involve the particles [33]. Nevertheless, values of 70 MPa can be achieved for the mortars prepared with CA. Looking at the mortars with CT, it can be observed that higher compressive strength is achieved, varying from 70 to 90 MPa, closer to the values obtained for the mortars with the commercial sand (W). Moreover, the compressive strength values begin to stabilize at the W/B ratio of 0.33, and the increase of about 22% in the water content (to a W/B ratio of 0.35) did not affect the strength of the material. Herewith, it is concluded that the optimum water content has already been achieved for the formulations MW0.29, MCA0.35 and MCT0.33 which are considered optimized.

The water absorption of the optimized samples is given in Table 4. The binder formulation has a lower water absorption value due to its compact matrix. MW0.29 mortar exhibits a similar value (0.77%) due to the water amount and granulometric distribution of the aggregate (commercial calibrated sand). The mortars MCA0.35 and MCT0.33 exhibit slightly higher absorption values (1.36 and 1.37%), due to the greater water content. Nevertheless, all the one-part AAMs prepared show suitable water absorption values (<5%), above which their compressive strength can be compromised [39]. In addition, the typical water absorption values for mortars with OPC vary between 15 and 18% [40], while for traditional AAMs they are slightly lower, from 5 to 11% [41,42].

**Figure 10:**
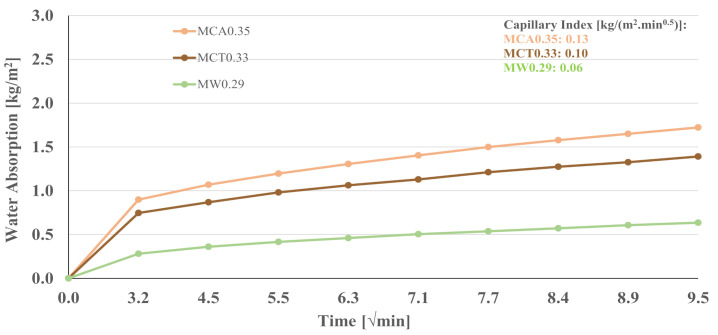
Capillary water absorption of optimized mortar samples, at 28 days of curing.

The results of the water absorption by capillary of the mortars are shown in Figure 10. The binder (B0.29) was also tested and the obtained capillary index value was 0.09 kg/(m^2^ min^0.5^).

The water absorption through capillary force is initially fast and becomes increasingly slower with time. Figure 10 shows that the MW0.29 mortar presents the lower cumulative water absorption, suggesting differences in the sample’s porosity. Figure 10 also reports the capillary index values for the investigated formulations. A direct relationship between this coefficient and the compressive strength is not clear, although the mortar showing the lowest strength presents the highest capillary index. Nevertheless, these values are considerably lower than those reported for mortars containing OPC—0.5 to 0.9 kg/(m^2^ min^0.5^) [43,44] and for AAM mortars, with 0.3 a 0.5 kg/(m^2^ min^0.5^) [42,43].

**Figure 11:**
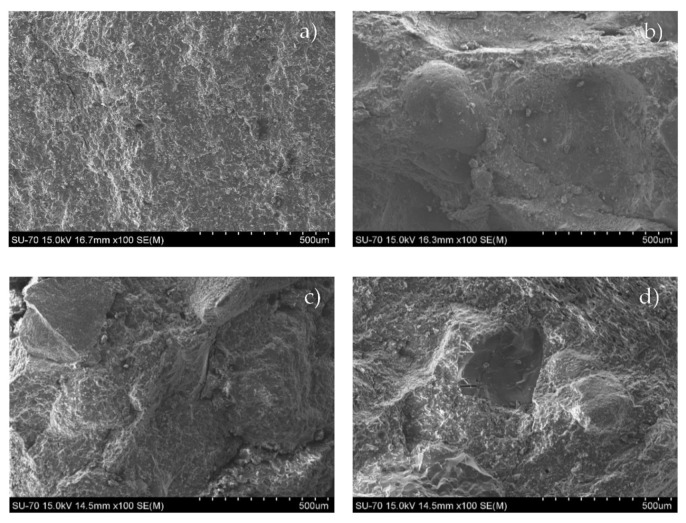
SEM micrographs of the binder, B0.29 (**a**) and mortars, MW0.29 (**b**); MCA0.35 (**c**); and MCT0.33 (**d**).

The morphology of the binder and mortars was evaluated by SEM micrograph—Figure 11.

B0.29 features a dense, homogeneous, and free of defects microstructure (Figure 11a). Mortars have a compact matrix, with a good incorporation of the aggregate into the binder in all tested formulations. Furthermore, no significant cracking or porosity (small micro-cracks resulting from samples preparation) are observed.

**Figure 12:**
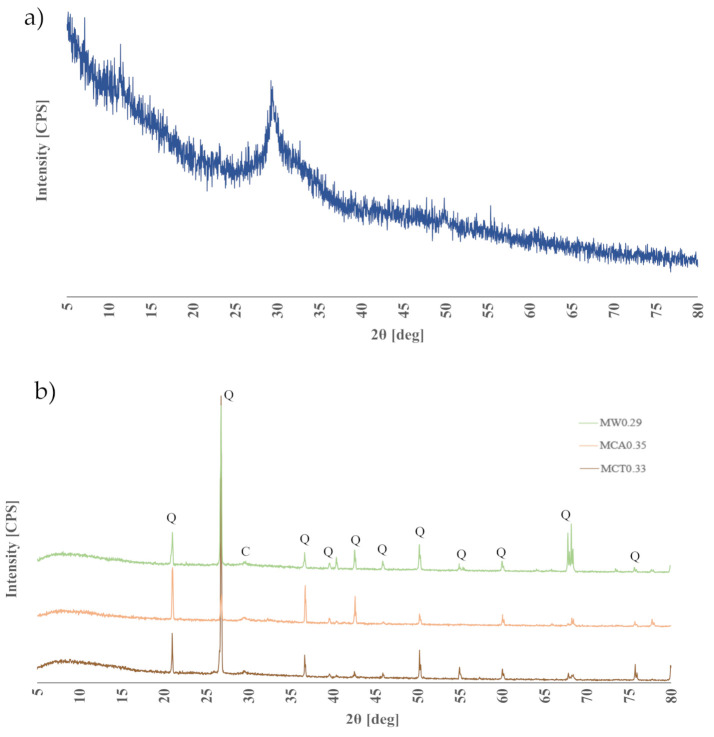
XRD patterns of the binder (**a**) and mortars (**b**) (Q—quartz; C—calcite).

XRD patterns of the optimized samples are shown in Figure 12. The binder has no noticeable crystalline phases since it is composed mostly of BFS and, for this reason, it can be considered an amorphous material [45]. As expected, all mortar patterns present visible peaks, namely quartz coming from the aggregates (sand). It is interesting to note the presence of calcite in all mortar samples, corresponding to the peak at around 29.5° 2θ and that MCA0.35 has the higher peak intensity, which agrees with the XRF results (Table 2). This is consistent with the obtained results in other studies that observe that the peak in this region results from the presence of calcium carbonate [37,46].

**Figure 13:**
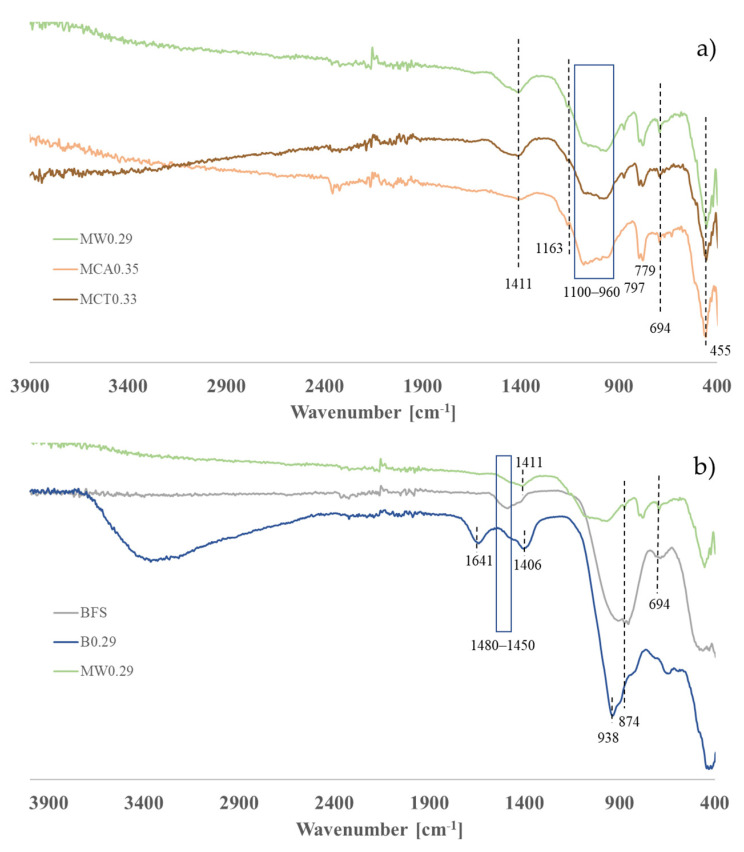
FTIR-ATR spectra of (**a**) mortars with the three aggregates (W, CA, and CT) and (**b**) BFS, binder (B0.29-BFS and sodium metasilicate) and mortar with commercial sand (MW0.29).

Figure 13 presents the FTIR-ATR spectra of the samples after 28 days of curing. The spectra of the one-part mortars with the three tested aggregates (W, CA and CT) are presented in Figure 13a and the comparison between BFS, binder (B0.29-BFS and sodium metasilicate) and the mortar with calibrated commercial sand (MW0.29) are patent in Figure 13b.

In Figure 13a,b at around 1440–1400 cm^−1^, a sharp valley is attributed to the presence of carbonates (CO_3_^2−^) formed upon curing, as well as the band at 874 cm^−1^. In the BFS, the band of O-C-O asymmetric stretching appears at 1480–1450 cm^−1^ indicating a possible carbonation of the raw material [47]. The characteristic band at 455 cm^−1^ present in all samples is attributed to O-Si-O bending vibrations. Anti-symmetric Si-O-T (T = Si or Al tetrahedral units) vibrations, assigned at 960–1100 cm^−1^, give additional information about the geopolymer structure in all AAMs (Figure 13a). Changes from 908 cm^−1^ in the BFS (Figure 13b) to 938 cm^−1^ in B0.29, and to 966 cm^−1^ in MW0.29 reveal polymerization degree enhancement. In the case of the mortars, the new band observed between 960 and 1100, which may result from a more extensive reaction of silicates and aluminosilicates present in the sands, justifying the lower mechanical resistance of the binder compared to that of mortars. In the BFS, this band appears at 908 cm^−1^ and this difference is attributed to structural differences of the raw material. The unreacted BFS presents one band at 694 cm^−1^, which is assigned to T-O asymmetric stretching vibration [6,17].

### 3.3. Comparative Analysis with Previous Works

Table 5 and Table 6 present a comparison of the main properties (fresh and hardened state characterization) of the materials obtained in this work and other one-part binders and mortars reported in the literature, respectively. From Table 5 it can be depicted that different precursors (i.e., fly ash, blast furnace slag, and rice husk) and solid activators (i.e., sodium silicate, sodium aluminate, sodium metasilicate) have been studied. Moreover, there are several works where AAMs are produced with two different activators [6,14,18]. The present work uses BFS and sodium metasilicate, formulation B0.29, and surpasses all other reported compressive strength values for one-part binders (56 MPa), except the work of Hajimohammadi and Deventer [14] that used fly ash, sodium silicate, and sodium hydroxide.

Table 6 shows that the formulation containing waste sand CT has one of the highest values for compressive strength ever reported for one-part AAM mortars (86 MPa), being surpassed only by a mortar using commercial sand [16]. Finally, the values attained for the mortar prepared with the calibrated commercial sand, W (79 MPa), are similar to those obtained by a formulation that contains a mixture of fly ash, BFS, and dune sand (84 MPa) [45]. CA waste sand led to a slight reduction in the mortar compressive strength (70 MPa) that is, however, higher than the reported in other works [10,45].

## 4. Conclusions

The main goal of this work was the development of waste-based one-part AAMs binders and mortars, cured at room temperature, with high compressive strength and adequate workability, using BFS as the precursor, sodium metasilicate as the solid activator, and waste sands as the aggregate.

A blast furnace slag based one-part AAM binder with a high compressive strength after 28 days of curing was developed. The attained mechanical resistance (56 MPa) surpasses all the values reported in the literature for one-part AAM binders activated using only sodium silicate.

It is possible to replace commercial sand (W) with exhausted sands from biomass boilers (CA and CT). Mortars prepared with waste sands need more water (higher W/B ratio) than those prepared with the commercial sand due to their smaller particle size (W/B ratio of 0.29, 0.33, and 0.35, respectively, for W, CT, and CA). Nevertheless, mortars with excellent mechanical properties were obtained.

The mortar prepared with CT waste sand exhibits one of the highest compressive strength values (86 MPa) ever reported for one-part AAM mortars. The mortar with CA has a slightly lower compressive strength (70 MPa) that is, however, higher than the values reported in the literature. The other evaluated properties (setting time, apparent density, and water absorption) confirm the feasibility of using CA and CT wastes as aggregates in AAMs mortars production.

Besides the outstanding compressive strength of these sustainable materials, this application will avoid the landfill deposition of the exhausted sand wastes reducing the environmental impact of the paper pulp industry. Given this last point, further studies are recommended involving the progressive replacement of BFS by local fly ash to observe the different effects on mortars performance.

## Figures and Tables

**Table 1:** One-part AAMs binder and mortars compositions.

Sample Name	Mixture Proportion [g]			W/B
	BFS	Sodium Metasilicate	Sand	
B0.29 B0.31 B0.33	100	10	-	0.29 0.31 0.33
MW0.29 MCA0.29 MCT0.29	100	10	200	0.29
MW0.33 MCA0.33 MCT0.33				0.33
MW0.35 MCA0.35 MCT0.35				0.35

**Table 2:** Chemical composition of precursor (BFS) and sands (W, CA, and CT), evaluated by XRF.

Component	BFS	W	CA	CT
	[wt.%]			
Na_2_O	0.29	0.07	1.69	0.42
MgO	6.77	0.06	2.16	0.69
Al_2_O_3_	**9.21**	1.15	2.86	3.83
SiO_2_	**32.43**	97.15	64.57	80.80
P_2_O_5_	0.01	0.02	1.03	0.34
SO_3_	1.81	-	0.55	0.05
Cl	0.02	-	0.22	0.08
K_2_O	0.55	0.79	2.01	2.03
CaO	**46.55**	0.11	20.00	7.30
TiO_2_	0.95	0.03	0.22	0.15
MnO	0.18	-	0.39	0.09
Fe_2_O_3_	0.36	0.09	1.24	1.54
Sr	0.09	-	0.05	-
Zr	0.04	-	-	-
Ba	0.06	-	0.03	0.01
**LOI**	**0.65**	**0.28**	**2.92**	**2.62**

**Table 3:** Apparent density of the mortar´s samples, at 7 and 28 days of curing.

Sample	Apparent Density [g/cm^3^]	
	7 days	28 days
MW0.29	2.20 ± 0.06	2.22 ± 0.03
MW0.33	2.16 ± 0.02	2.14 ± 0.05
MW0.35	2.14 ± 0.19	2.15 ± 0.09
MCA0.29	1.98 ± 0.08	1.94 ± 0.07
MCA0.33	1.84 ± 0.24	1.85 ± 0.23
MCA0.35	2.11 ± 0.06	2.10 ± 0.06
MCT0.29	2.09 ± 0.11	2.09 ± 0.13
MCT0.33	2.10 ± 0.05	2.07 ± 0.05
MCT0.35	2.15 ± 0.05	2.07 ± 0.01

**Table 4:** Water absorption by total immersion, for optimized samples, at 28 days of curing.

Sample	Water Absorption [%]
B0.29	0.70 ± 0.04
MW0.29	0.77 ± 0.03
MCA0.35	1.36 ± 0.03
MCT0.33	1.37 ± 0.15

**Table 5:** Main properties of several one-part binders reported in literature (at 28 days of curing).

Precursors	Solid Activator	W/B	Curing Condition	Workability [mm]	Final Setting Time [min]	Apparent Density [kg/m^3^]	R_c_^(+)^ [MPa]	Water Abs. [%]	Ref.
FA	Na_2_SiO_3_ and NaOH	0.34	40 °C	-	-	-	65	-	[14]
**BFS**	**Na_2_SiO_3_**	**0.29**	**Ambient**	**114**	**140**	**1820**	**56**	**0.7**	**This work**
FA	Na_2_SiO_3_	0.25	25 °C	241	70	2130	50	-	[15]
FA and BFS	Na_2_SiO_3_	0.37	23 °C	164	255	-	45	-	[21]
FA and BFS	Na_2_SiO_3_	0.3	23 °C 70%RH	240	-	-	30	-	[5]
Rice husk	NaAlO_2_	0.5	80 °C 80%RH	-	-	-	30^(*)^	-	[17]
FA and slag	Na_2_SiO_3_ and Ca(OH)_2_	0.45	Ambient	200	-	1730	27	-	[6]
FA and slag	Na_2_SiO_3_	0.30	Ambient	225	-	1760	24	-	[6]
FA	Na_2_SiO_3_	0.30	40 °C	-	-	-	21	-	[14]
FA	NaOH and Na_2_SiO_3_	0.40	20 °C	-	-	-	11	-	[18]

^(*)^ 7 days testing; ^(+)^ Compressive strength.

**Table 6:** Main properties of several one-part mortars reported in literature (at 28 days of curing).

Precursors	Solid Activator	W/B	Curing Condition	Aggregate	Workability [mm]	Final Setting Time [min]	Apparent Density [kg/m^3^]	R_c_^(+)^ [MPa]	Water Abs. [%]	Ref.
BFS	Na_2_SiO_3_	0.34	22 °C 100%RH	Sand	-	30	-	105	-	[16]
**BFS**	**Na_2_SiO_3_**	**0.33**	**Ambient**	**Waste sand (CT)**	**123**	**120**	**1950**	**86**	**1.37**	**This work**
FA and BFS	Na_2_SiO_3_	0.31	23 °C 95%RH	Dune sand	-	-	-	84	-	[45]
**BFS**	**Na_2_SiO_3_**	**0.29**	**Ambient**	**Sand**	**120**	**110**	**2220**	**79**	**0.77**	**This work**
**BFS**	**Na_2_SiO_3_**	**0.35**	**Ambient**	**Waste sand (CA)**	**123**	**130**	**1990**	**70**	**1.36**	**This work**
BFS and microsilica	Na_2_SiO_3_	0.34	23 °C 95%RH	Dune sand	-	-	-	43	-	[45]
Kaolin, FA and OPC	Ca(OH)_2_	0.29	58%RH	Sand	-	-	-	43	-	[10]

^(+)^ Compressive strength.

## Data Availability

Data sharing is not applicable to this article.

## References

[1] Huntzinger D.N., Eatmon T.D. (2009). A life-cycle assessment of Portland cement manufacturing: Comparing the traditional process with alternative technologies. J. Clean. Prod..

[2] Eurostat: Statistics Explained Climate Change—Driving Forces 2020. https://ec.europa.eu/eurostat/statistics-explained/index.php?title=Climate_change_-_driving_forces.

[3] Assi L., Carter K., Deaver E., Anay R., Ziehl P. (2018). Sustainable concrete: Building a greener future. J. Clean. Prod..

[4] Reddy M.S., Dinakar P., Rao B.H. (2018). Mix design development of fly ash and ground granulated blast furnace slag based geopolymer concrete. J. Build. Eng..

[5] Oderji S.Y., Chen B., Ahmad M.R., Shah S.F.A. (2019). Fresh and hardened properties of one-part fly ash-based geopolymer binders cured at room temperature: Effect of slag and alkali activators. J. Clean. Prod..

[6] Askarian M., Tao Z., Samali B., Adam G., Shuaibu R. (2019). Mix composition and characterisation of one-part geopolymers with different activators. Constr. Build. Mater..

[7] Koloušek D., Brus J., Urbanova M., Andertova J., Hulinsky V., Vorel J. (2007). Preparation, structure and hydrothermal stability of alternative (sodium silicate-free) geopolymers. J. Mater. Sci..

[8] Yang K.H., Song J.K., Ashour A.F., Lee E.T. (2008). Properties of cementless mortars activated by sodium silicate. Constr. Build. Mater..

[9] Luukkonen T., Abdollahnejad Z., Yliniemi J., Kinnunen P., Illikainen M. (2018). One-part alkali-activated materials: A review. Cem. Concr. Res..

[10] Abdollahnejad Z., Pacheco-Torgal F., de Aguiar J.B. Eco-concrete: One-part geopolymer mixes. Proceedings of the TRF Senior Research Scholars Progress II.

[11] Garcia-Lodeiro I., Fernández-Jimenez A., Palomo A. (2015). Cements with a low clinker content: Versatile use of raw materials. J. Sustain. Cem. Mater..

[12] Askarian M., Tao Z., Adam G., Samali B. (2018). Mechanical properties of ambient cured one-part hybrid OPC-geopolymer concrete. Constr. Build. Mater..

[13] Nematollahi B., Sanjayan J. (2017). Ambient temperature cured one-part engineered geopolymer composite: A sustainable alternative to engineered. Constr. Build. Mater..

[14] Hajimohammadi A., van Deventer J.S.J. (2017). Characterisation of one-part geopolymer binders made from fly ash. Waste Biomass Valorization.

[15] Mohammed B.S., Haruna S., Wahab M.M.A., Liew M.S., Haruna A. (2019). Mechanical and microstructural properties of high calcium fly ash one-part geopolymer cement made with granular activator. Heliyon.

[16] Luukkonen T., Sreenivasan H., Abdollahnejad Z., Yliniemi J., Kantola A., Telkki V.V., Kinnunen P., Illikainen M. (2020). Influence of sodium silicate powder silica modulus for mechanical and chemical properties of dry-mix alkali-activated slag mortar. Constr. Build. Mater..

[17] Sturm P., Gluth G.J.G., Brouwers H.J.H., Kühne H.C. (2016). Synthesizing one-part geopolymers from rice husk ash. Constr. Build. Mater..

[18] Suwan T., Fan M. (2017). Effect of manufacturing process on the mechanisms and mechanical properties of fly ash-based geopolymer in ambient curing temperature. Mater. Manuf. Process..

[19] Nematollahi B., Sanjayan J., Shaikh F.U.A. (2015). Synthesis of heat and ambient cured one-part geopolymer mixes with different grades of sodium silicate. Ceram. Int..

[20] Oderji S.Y., Chen B., Shakya C., Ahmad M.R., Shah S.F.A. (2019). Influence of superplasticizers and retarders on the workability and strength of one-part alkali-activated fly ash/slag binders cured at room temperature. Constr. Build. Mater..

[21] Bong S.H., Nematollahi B., Nazari A., Xia M., Sanjayan J. (2019). Efficiency of different superplasticizers and retarders on properties of “one-part” fly ash-slag blended geopolymers with different activators. Materials.

[22] Torres A., Brandt J., Lear K., Liu J. (2017). A looming tragedy of the sand commons. Science.

[23] Gholampour A., Ho V.D., Ozbakkaloglu T. (2019). Ambient-cured geopolymer mortars prepared with waste-based sands: Mechanical and durability-related properties and microstructure. Compos. Part B Eng..

[24] Norma Portuguesa (2003). NP EN 1097-6: Methods of Test for Aggregates, Part 6: Bulk Density and Water Absorption.

[25] Norma Portuguesa (2000). NP EN 933-1: Methods of Test for Aggregates, Part 1: Sieve Analysis.

[26] CEN: European Committee for Standardization (1998). EN 1015-3: Methods of Test for Mortar for Masonry Part 3—Determination of Consistence of Fresh Mortar (by Flow Table).

[27] CEN: European Committee for Standardization (2005). EN 196-3: Methods of Testing Cement Part 3—Determination of Setting Times and Soundness.

[28] CEN: European Committee for Standardization (1999). EN 1015-11: Methods of Test for Mortar for Masonry Part 11—Determination of Flexural and Compressive Strength of Hardened Mortar.

[29] CEN: European Committee for Standardization (2002). EN 1015-18: Methods of Test for Mortar for Masonry Part 18—Determination of water- Absorption Coefficient Due to Capillary Action of Hardened Mortar.

[30] Pourkhorshidi A.R., Najimi M., Parhizkar T., Jafarpour F., Hillemeier B. (2010). Applicability of the standard specifications of ASTM C618 for evaluation of natural pozzolans. Cem. Concr. Compos..

[31] Pangdaeng S., Phoo-ngernkham T., Sata V., Chindaprasirt P. (2014). Influence of curing conditions on properties of high calcium fly ash geopolymer containing Portland cement as additive. Mater. Des..

[32] Suwan T., Fan M. (2014). Influence of OPC replacement and manufacturing procedures on the properties of self-cured geopolymer. Constr. Build. Mater..

[33] Venkatarama Reddy B.V., Gupta A. (2008). Influence of sand grading on the characteristics of mortars and soil-cement block masonry. Constr. Build. Mater..

[34] Criado M., Walkley B., Ke X., Provis J.L., Bernal S.A. (2018). Slag and activator chemistry control the reaction kinetics of sodium metasilicate-activated slag cements. Sustainability.

[35] Bernal S.A., Provis J.L., Rose V., De Gutierrez R.M. (2011). Evolution of binder structure in sodium silicate-activated slag-metakaolin blends. Cem. Concr. Compos..

[36] Puligilla S., Mondal P. (2013). Role of slag in microstructural development and hardening of fly ash-slag geopolymer. Cem. Concr. Res..

[37] Luukkonen T., Abdollahnejad Z., Yliniemi J., Kinnunen P., Illikainen M. (2018). Comparison of alkali and silica sources in one-part alkali-activated blast furnace slag mortar. J. Clean. Prod..

[38] Singh S.B., Munjal P., Thammishetti N. (2015). Role of water/cement ratio on strength development of cement mortar. J. Build. Eng..

[39] Yahya Z., Abdullah M.M.A.B., Talib S.Z.A., Razak R.A. Comparative study on early strength of sodium hydroxide (NaOH) activated fly ash based geopolymer. Proceedings of the AIP Conference.

[40] Gulbe L., Vitina I., Setina J. (2017). The influence of cement on properties of lime mortars. Procedia Eng..

[41] Mermerdaş K., Manguri S., Nassani D.E., Oleiwi S.M. (2017). Effect of aggregate properties on the mechanical and absorption characteristics of geopolymer mortar. Eng. Sci. Technol. Int. J..

[42] Novais R.M., Carvalheiras J., Senff L., Labrincha J.A. (2018). Upcycling unexplored dregs and biomass fly ash from the paper and pulp industry in the production of eco-friendly geopolymer mortars: A preliminary assessment. Constr. Build. Mater..

[43] Mobili A., Telesca A., Marroccoli M., Tittarelli F. (2020). Calcium sulfoaluminate and alkali-activated fly ash cements as alternative to Portland cement: Study on chemical, physical-mechanical, and durability properties of mortars with the same strength class. Constr. Build. Mater..

[44] Deboucha W., Leklou N., Khelidj A., Oudjit M.N. (2017). Natural pozzolana addition effect on compressive strength and capillary water absorption of Mortar. Energy Procedia.

[45] Dong M., Elchalakani M., Karrech A. (2020). Development of high strength one-part geopolymer mortar using sodium metasilicate. Constr. Build. Mater..

[46] Samantasinghar S., Singh S.P. (2018). Effect of synthesis parameters on compressive strength of fly ash-slag blended geopolymer. Constr. Build. Mater..

[47] Gao X., Yu Q.L., Brouwers H.J.H. (2015). Reaction kinetics, gel character and strength of ambient temperature cured alkali activated slag-fly ash blends. Constr. Build. Mater..

